# Identification of SORT1 as a Key Regulator of Antigen Presentation in CD16+ Monocytes During Septic Shock via Multi‐Omics and In Silico Gene Knockout Analysis

**DOI:** 10.1002/iid3.70548

**Published:** 2026-10-06

**Authors:** Shulu Wu, Jiaying Zhang, Shiqi Guo, Yuting Hu, Hui Shen, Yingjie Xu, Bining Yang

**Affiliations:** ^1^ Department of Emergency and Critical Care Medicine The Fourth Affiliated Hospital of Soochow University, Suzhou Dushu Lake Hospital Suzhou China

**Keywords:** CD16+ monocytes, machine learning, septic shock, single‐cell transcriptomics, SORT1

## Abstract

**Objective:**

This study aimed to screen and identify hub genes correlated with septic shock through integrative analyses of bulk transcriptomics, machine learning, weighted gene co‐expression network analysis (WGCNA), single‐cell transcriptomics, and in silico gene knockout, and further dissect their underlying immunoregulatory mechanisms.

**Methods:**

Multiple bulk transcriptomic datasets of septic shock were integrated and processed via batch correction and differential expression analysis, followed by WGCNA. Candidate genes were screened by intersecting disease‐related differentially expressed genes, module genes derived from WGCNA, and predicted drug/compound targets. A diagnostic classifier was established by integrating 113 machine learning algorithms, with its predictive performance assessed using a training cohort and an independent external validation cohort. SHAP analysis was implemented to quantify and interpret the feature importance of model biomarkers. Single‐cell transcriptomic data from the dataset GSE175453 were subsequently used to characterize the cell‐type localization of hub genes, and scTenifoldKnk was adopted to conduct in silico gene knockout experiments on pivotal genes. Finally, gene set enrichment analysis (GSEA) based on Z‐score ranking was performed to dissect the underlying regulatory signaling pathways of these hub genes.

**Results:**

Batch correction markedly optimized the distribution of samples from distinct datasets. Combined differential expression analysis and WGCNA identified prominent transcriptional perturbations associated with immunity and inflammation in septic shock. Multidimensional intersection screening yielded candidate hub genes, from which core genes including *SORT1, Turquoise, blue, and brown modules IL18RAP, PLEKHA1, SLC38A1*, and *LDHA* were prioritized. Machine learning evaluations demonstrated that diagnostic models constructed from these candidate genes exhibited strong discriminatory capacity in both training and independent external validation cohorts. Notably, the RF + SVM hybrid model yielded AUC values of 0.998, 0.986 and 0.972 for the training set, GSE137340 and GSE26440, respectively. SHAP analysis verified that *SORT1* exerted the largest predictive contribution to the classification model. Single‐cell transcriptomic profiling illustrated that *SORT1* was predominantly enriched in monocytes, especially the CD16+ monocyte subset, with significantly elevated expression in patients with septic shock. In silico gene knockout of *SORT1* led to pronounced transcriptional dysregulation of *B2M, CD74* and multiple *HLA* class II family genes. Gene set enrichment analysis (GSEA) performed on Z‐score ranked genome‐wide transcripts further uncovered robust enrichment of biological pathways governing antigen processing and presentation, MHC class II antigen presentation, as well as interferon‐mediated immune responses.

**Conclusion:**

*SORT1* represents a promising hub gene for the diagnosis and mechanistic investigation of septic shock. Its upregulation in CD16+ monocytes, combined with the pronounced transcriptional dysregulation of antigen presentation‐associated genes upon in silico *SORT1* knockout, indicates that *SORT1* may drive immune dysregulation during septic shock by modulating the antigen‐presenting phenotype of CD16+ monocytes.

## 1 Introduction

Septic shock represents the most severe manifestation of sepsis, defined by profound circulatory, cellular, and metabolic derangements, with its pathogenesis centered on infection‐triggered dysregulated host response and secondary organ dysfunction [1]. Per the Sepsis‐3 international consensus, septic shock requires vasoactive agents to maintain a mean arterial pressure ≥ 65 mmHg despite adequate fluid resuscitation, accompanied by serum lactate levels > 2 mmol/L, conferring a substantially higher mortality risk than uncomplicated sepsis [2]. Global Burden of Disease data demonstrate that sepsis continues to impose a massive public health burden worldwide, with high morbidity and mortality [3]. Despite steady advances in anti‐infective therapy, fluid resuscitation, vasopressor management, and organ support, the mortality rate of septic shock remains persistently elevated [4]. A core reason is that septic shock is not merely a hyperinflammatory state, but a complex immune homeostatic disorder featuring concurrent or interleaved pro‐inflammation, immunosuppression, immune cell exhaustion, and metabolic dysregulation [5, 6, 7, 8]. Patients typically show early massive release of inflammatory mediators, along with monocyte functional reprogramming, downregulated MHC‐II‐associated antigen presentation, and aberrant interferon responses; persistently low monocyte HLA‐DR expression is also strongly linked to secondary infection and poor prognosis [9, 10, 11]. Conventional biomarkers such as procalcitonin and interleukin‐6 partially reflect infection severity and systemic inflammation, but their diagnostic and prognostic performance varies across populations and lacks immune cell subset specificity, limiting their value for immune phenotyping and mechanistic analysis [12, 13]. Thus, there is an urgent need to identify novel molecular markers with diagnostic utility, cell‐type specificity, and immunomodulatory relevance.

Monocytes are critical effector cells of the innate immune system, and undergo profound quantitative, phenotypic, and functional remodeling during septic shock. Based on the surface expression levels of CD14 and CD16, human peripheral blood monocytes are conventionally classified into three subsets: classical CD14 + + CD16‐ monocytes, intermediate CD14 + + CD16+ monocytes, and non‐classical CD14 + CD16 + + monocytes [14]. In single‐cell transcriptomic studies, intermediate and non‐classical monocytes are often collectively termed CD16+ monocytes (CD16+ Mono) based on clustering features, despite substantial functional differences between the two subsets. Intermediate CD16+ monocytes typically show high expression of HLA‐DR, HLA‐DP, HLA‐DQ and antigen processing‐related molecules, endowing them with robust MHC‐II‐restricted antigen‐presenting capacity [15, 16]. Non‐classical CD16+ monocytes are characterized by endothelial patrolling, adhesion and transendothelial migration, and participate in inflammatory tissue recruitment and vascular injury via the CX3CR1–CX3CL1 axis [17].

In patients with sepsis, monocyte subset composition and transcriptional profiles undergo extensive alterations. Single‐cell studies have identified multiple disease‐associated monocyte states, including sepsis‐specific monocytes with unique transcriptional signatures and HLA‐DR^low^S100A^high^ immunosuppressive monocytes [18, 19]. These findings indicate that monocyte dysfunction in sepsis extends beyond numerical changes, and involves profound remodeling of antigen presentation, inflammatory response, and immunosuppressive programs. Nevertheless, the key molecules that regulate the antigen‐presenting function and immune state transition of CD16+ monocytes remain poorly characterized, which limits in‐depth mechanistic understanding of monocyte immune dysregulation in septic shock.

High‐throughput transcriptomic technology serves as a core tool for dissecting the molecular heterogeneity and immune regulatory networks of septic shock. Bulk transcriptomics can profile global gene expression at the tissue or cell population level, yet its detection results are confounded by both intracellular expression shifts and alterations in cellular composition, making it difficult to directly pinpoint the cellular origins of candidate genes. Weighted Gene Co‐expression Network Analysis (WGCNA) constructs co‐expression modules based on gene expression correlations and screens core network nodes strongly associated with disease phenotypes [20]. Machine learning algorithms can mine feature combinations with stable classification performance from high‐dimensional representation data, and the SHapley Additive exPlanations (SHAP) method can quantify the contribution of each feature to model outputs at both the global and single‐sample levels, effectively improving the interpretability of complex machine learning models. Single‐cell transcriptome sequencing can accurately resolve the transcriptional heterogeneity of various immune cells and their subpopulations, and identify the main cell subpopulations where candidate genes are expressed as well as disease‐related expression patterns [21, 22]. In silico gene knockout tools represented by scTenifoldKnk can simulate the network perturbation effects after target gene deletion based on single‐cell gene regulatory networks, providing computational evidence for the prioritized screening of downstream genes and functional pathways [23]. In summary, by integrating bulk transcriptomics, WGCNA, interpretable machine learning, single‐cell transcriptomics and in silico gene perturbation analysis, a multi‐level research system covering disease‐related gene screening, diagnostic value evaluation, cell localization and potential mechanism prediction can be constructed.

Sortilin 1, encoded by the *SORT1* gene, is a transmembrane sorting receptor belonging to the VPS10P domain family. It is predominantly localized to the trans‐Golgi network and endosomal system, with a small fraction present on the plasma membrane, and mediates protein endocytosis, vesicular sorting, and intracellular trafficking. Accumulating evidence has demonstrated that sortilin regulates the Golgi‐endosome‐plasma membrane trafficking of immune effector molecules such as interferon‐γ [24], and participates in the delivery of lysosomal proteins from the Golgi apparatus to phagosomes as well as phagosome maturation in macrophages [25]. In addition, sortilin is highly expressed in macrophages and dendritic cells, and its deficiency impairs the antigen processing capacity of dendritic cells and the ability of macrophages to clear intracellular pathogens [26, 27]. These findings suggest that *SORT1* may serve as a key molecule linking intracellular vesicular trafficking, antigen processing, cytokine secretion, and myeloid cell immune function. However, the diagnostic potential and cell subset‐specific expression pattern of *SORT1* in septic shock, as well as whether it participates in regulating the antigen presentation network of CD16+ monocytes, remain largely unexplored. Therefore, this study aims to identify key genes associated with septic shock by integrating bulk transcriptomics, machine learning, single‐cell transcriptomics, and in silico gene knockout analysis, with a focus on exploring the potential regulatory role of *SORT1* in immune functional remodeling of CD16+ monocytes.

## 2 Materials and Methods

### 2.1 Data Sources and Research Design

Transcriptomic datasets associated with septic shock were retrieved from the public Gene Expression Omnibus (GEO) database. For bulk transcriptomic profiling, datasets GSE57065 and GSE95233 were integrated to form a training cohort, which was applied for differential expression analysis, weighted gene co‐expression network analysis (WGCNA), candidate gene screening, and construction of diagnostic machine learning models. Datasets GSE26440 and GSE137340 served as independent external validation cohorts to evaluate the generalization performance of the established classifiers. Samples were stratified into control and septic shock groups according to the clinical metadata provided within each dataset.

The comprehensive analytical pipeline consisted of bulk transcriptome preprocessing, batch effect correction, differential expression analysis, weighted gene co‐expression network analysis, candidate gene identification, functional enrichment profiling, machine learning model construction and external validation, SHapley Additive exPlanations (SHAP)‐based model interpretation, single‐cell transcriptomic profiling, cell‐type localization of hub genes, antigen presentation scoring, in silico gene knockout, and Z‐score‐ranked gene set enrichment analysis (GSEA). The single‐cell dataset GSE175453 was further adopted to characterize the cell‐type‐specific expression patterns and biological functions of hub genes, as well as to conduct subsequent mechanistic investigations.

### 2.2 Bulk Transcriptome Data Preprocessing

Expression matrices and matched platform annotation files of GSE57065, GSE95233, GSE26440 and GSE137340 were downloaded from the GEO database. Probe IDs were first converted to official gene symbols according to platform annotations. For genes matched by multiple probes, the mean expression value of all corresponding probes was assigned as the final expression level of the target gene. Each dataset's expression matrix was then normalized, and only genes shared across all datasets were retained for downstream analyses.

For the training cohort, expression matrices of GSE57065 and GSE95233 were integrated. Owing to disparities in sequencing platforms, experimental batches and sample origins across independent datasets, the combined training matrix was subjected to batch effect correction. Principal component analysis (PCA) was conducted pre‐ and post‐correction to assess the efficacy of batch elimination by visualizing sample clustering patterns in low‐dimensional space. Datasets GSE26440 and GSE137340 were processed as independent external validation cohorts following identical gene annotation and normalization pipelines applied to the training set; only genes overlapping with the training cohort were preserved for subsequent model validation.

### 2.3 Differential Expression Analysis

Differential expression analysis was implemented to compare transcriptomic profiles between control and septic shock samples within the training cohort. Linear models combined with empirical Bayesian moderation were adopted to stabilize the estimation of gene expression variances. A design matrix was generated according to sample grouping metadata, and intergroup expression fold changes were calculated for every gene. Differentially expressed genes (DEGs) were filtered by thresholds of adjusted *p*‐values and expression fold changes. Volcano plots were generated to visualize the global distribution of DEGs, while heatmaps were plotted to exhibit the expression signatures of representative DEGs across all samples, which further assessed the discriminatory capacity of these DEGs to separate control subjects from patients with septic shock.

### 2.4 Weighted Gene Co‐Expression Network Analysis

Weighted gene co‐expression network analysis (WGCNA) was performed on the training cohort to identify co‐expression modules tightly correlated with the septic shock phenotype. The expression matrix was preprocessed to filter out low‐variance genes, and sample clustering was applied to detect potential outlier samples. An optimal soft threshold was determined according to the scale‐free topology fit index, which enabled construction of a weighted gene co‐expression network satisfying scale‐free distribution properties.

A topological overlap matrix was derived from the weighted adjacency matrix, and gene co‐expression modules were delineated via hierarchical clustering coupled with dynamic tree cutting. Each module was summarized by its module eigengene, followed by correlation analysis between module eigengenes and the septic shock phenotype. Modules exhibiting significant phenotypic correlations were designated as disease‐relevant modules. Module membership and gene significance metrics were subsequently quantified to evaluate the correlation strength of intra‐module genes with the disease phenotype, and all genes harbored within disease‐associated modules were retained for downstream candidate gene screening.

### 2.5 Candidate Gene Screening

Multi‐source gene sets encompassing differentially expressed genes (DEGs), genes within WGCNA disease‐relevant modules, and candidate therapeutic targets predicted from external databases or preliminary analyses were integrated to screen candidate genes with robust diagnostic capacity and biological relevance. Intersection analysis was performed to extract overlapping genes shared across all gene collections, and Venn diagrams were generated to visualize the overlapping relationships among distinct gene sets. Genes obtained from the intersection were designated as candidate genes for subsequent functional enrichment and machine learning model construction. Hub genes were finally prioritized by synthesizing three lines of evidence: model predictive performance, SHapley Additive exPlanations (SHAP) feature importance, and cell‐type expression patterns derived from single‐cell transcriptomic profiling.

### 2.6 GO and KEGG Enrichment Analysis

Gene Ontology (GO) and Kyoto Encyclopedia of Genes and Genomes (KEGG) enrichment analyses were conducted to annotate the biological functions of candidate genes. Briefly, all gene symbols were converted to Entrez IDs prior to enrichment of GO biological processes and KEGG signaling pathways. Enriched terms were sorted by adjusted *p* values, and bubble plots, lollipop plots and bar graphs were generated to visualize top enriched functional categories. We particularly focused on pathways associated with immunoregulation, inflammatory response, leukocyte activation, antigen processing and presentation, cytokine signaling, NF‐κB cascade, and metabolic reprogramming.

### 2.7 Machine Learning Model Construction and External Validation

Diagnostic classifiers for septic shock were built using the expression profiles of candidate genes derived from the training cohort, which integrated datasets GSE57065 and GSE95233. To screen classifiers with robust predictive performance and satisfactory cross‐dataset generalization, a comprehensive panel of machine learning algorithms and hybrid combinatorial models were compared, including LASSO, Ridge regression, Elastic Net, random forest, support vector machine, gradient boosting, XGBoost, linear discriminant analysis, and naive Bayes.

All models were trained and subjected to feature selection within the training cohort, and their discriminative capacity was assessed via receiver operating characteristic (ROC) curves, with predictive efficacy quantified by the area under the curve (AUC). Datasets GSE26440 and GSE137340 served as independent external validation cohorts to further test the generalization capacity of each classifier across distinct sample batches. AUC values were computed individually for the training cohort and each validation cohort, and an AUC heatmap was generated to systematically compare the overall predictive performance of all combinatorial models. Classifiers exhibiting stable and high discriminatory power in both training and external validation cohorts were designated as the optimal diagnostic models.

### 2.8 SHAP Model Explanation and Key Gene Screening

SHAP analysis was implemented to quantify the feature importance of each candidate gene and clarify their respective contributions to the predictive outputs of machine learning classifiers. Candidate genes were ranked according to their mean absolute SHAP values to pinpoint hub genes with dominant predictive effects. SHAP summary plots were generated to visualize the global influence of gene expression abundance on model classification results, whereas SHAP dependence plots were applied to characterize the correlation between expression variation of individual genes and predictive outcomes. Finally, multi‐dimensional evidence encompassing model discriminatory performance, SHAP feature weights, feature stability and biological interpretability was integrated to prioritize hub genes including SORT1 for subsequent single‐cell transcriptomic profiling and mechanistic exploration.

### 2.9 Single‐Cell Transcriptome Data Processing

The single‐cell transcriptomic dataset GSE175453 was utilized to dissect the cellular source and biological functions of hub genes in septic shock. Raw 10× Genomics expression matrices were imported, and only the Gene Expression matrix was retained for transcriptomic analysis, excluding the Antibody Capture matrix provided in this dataset.

Individual Seurat objects were generated for each sample, followed by quality control filtering based on the number of detected genes, total UMI counts, mitochondrial gene percentage and hemoglobin gene proportion. Low‐quality cells were discarded, and DoubletFinder was applied to detect and eliminate putative doublets. Processed samples were integrated into a merged Seurat object, which underwent normalization, highly variable gene identification, scaling and principal component analysis sequentially. The Harmony algorithm was adopted to mitigate batch biases derived from distinct sample sources. A shared nearest‐neighbor graph was built on Harmony‐corrected low‐dimensional embeddings for cell clustering, with cellular heterogeneity visualized via UMAP and t‐SNE dimensional reduction.

### 2.10 Cell Type Annotation

Cell clusters were manually annotated using canonical cell‐type marker genes. The annotated major immune populations comprised CD14+ monocytes, CD16+ monocytes, CD4 + T cells, CD8 + T cells, natural killer (NK) cells, B cells, plasma cells, megakaryocytes, dendritic cells (DCs), and hematopoietic stem and progenitor cells (HSPCs).

CD14+ monocytes were characterized by marker genes LYZ, CD14, FCN1 and S100A8; CD16+ monocytes were defined by FCGR3A, MS4A7 and LST1; pan‐T cell markers CD3D, CD3E and IL7R, together with CD8A and CD8B, distinguished T cell subsets. NK cells were identified via NKG7, GNLY and PRF1; B cells by MS4A1 and CD79A; plasma cells by MZB1, XBP1 and JCHAIN; megakaryocytes by PPBP, PF4 and TUBB1; DCs by CD1C, FCER1A and LILRA4.

Annotation reliability was verified with UMAP embeddings, marker dot plots and feature expression distribution maps of signature genes.

### 2.11 Expression Analysis of Key Genes at the Single‐Cell Level

FeaturePlot, VlnPlot and DotPlot functions were applied to visualize cell‐type‐specific expression signatures of hub genes including SORT1, so as to delineate their distribution across distinct immune subsets. Single‐cell profiling revealed that SORT1 was predominantly enriched in monocytes, with the highest abundance restricted to CD16+ monocytes; accordingly, downstream analyses centered on SORT1 expression alterations within this monocyte subpopulation. The Wilcoxon rank‐sum test was performed to quantify intergroup expression differences of SORT1 in CD16+ monocytes between healthy controls and patients with septic shock, and violin plots paired with box plots were generated to exhibit the corresponding expression distributions.

### 2.12 In Silico Gene Knockout Analysis

scTenifoldKnk was utilized for in silico knockout of SORT1 to characterize its regulatory functions within CD16+ monocytes isolated from septic shock patients. CD16+ monocyte RNA count matrices were extracted from the fully annotated integrated single‐cell dataset. Highly variable genes were identified and merged with SORT1 to form the input gene panel. All expression matrices were converted to data frames before downstream perturbation analysis to meet the format requirements of scTenifoldKnk.

SORT1 was set as the targeted gene for computational ablation. Multiple single‐cell gene regulatory networks were reconstructed to simulate transcriptional network disturbances upon SORT1 knockout, yielding differential regulation statistics for all profiled genes. The output diffRegulation table was retrieved, and genes with significant regulatory shifts were filtered using adjusted *p*‐values. Volcano plots and top‐ranked gene bar graphs were generated to visualize transcripts exhibiting the most profound transcriptional perturbation after SORT1 in silico knockout.

### 2.13 GSEA Analysis

Given the limited number of significantly perturbed genes identified via in silico knockout, gene set enrichment analysis (GSEA) was further performed on all genes ranked by their network perturbation Z‐scores. Gene names and matched Z‐score values were extracted from scTenifoldKnk outputs, after which entries containing missing or infinite values were discarded. For duplicated gene entries, records with the maximum absolute Z‐score were preserved. The final ranked gene list for GSEA was generated by sorting all genes in descending order of Z‐scores.

GSEA was performed using Hallmark, GO‐BP, and KEGG gene sets. Enrichment outputs were interpreted with reference to normalized enrichment scores (NES) and adjusted *p*‐values. Particular attention was paid to pathways governing antigen processing and presentation, MHC class II antigen presentation, interferon response, TNF‐α/NF‐κB signaling cascade, complement activation, and inflammatory signaling. Enrichment curves were generated to exhibit representative pathways, whereas lollipop plots of NES values were constructed to visualize global enrichment profiles.

### 2.14 Software and Reproducibility

All bioinformatic analyses were implemented within R version 4.3.2. Dedicated R packages were applied to execute each analytical workflow, including differential expression testing, batch effect correction, co‐expression network construction, functional enrichment profiling, machine learning classifier training, single‐cell transcriptomic processing, and in silico gene knockout simulation. The core packages utilized in this study consisted of limma, sva, WGCNA, clusterProfiler, org.Hs.eg.db, msigdbr, caret, glmnet, randomForest, e1071, xgboost, gbm, pROC, Seurat, harmony, DoubletFinder, scTenifoldKnk, ggplot2, pheatmap and ComplexHeatmap. Detailed version information for all primary R packages was summarized in Supplementary Table S1 to guarantee full reproducibility of the analytical pipeline.

### 2.15 Statistical Analysis

All statistical computations were conducted in R. The Wilcoxon rank‐sum test was adopted for intergroup comparisons of continuous variables. Multiple testing correction for differential expression *p*‐values was implemented via the Benjamini–Hochberg procedure. Adjusted *p*‐values and false discovery rates (FDR) served as the significance metrics for GO, KEGG, and GSEA enrichment outputs. Receiver operating characteristic (ROC) curves and corresponding area under the curve (AUC) values were applied to quantify the discriminative performance of diagnostic classifiers. Unless otherwise stated, statistical significance was defined as an adjusted *p*‐value < 0.05.

## 3 Results

### 3.1 Data Integration, Differential Expression Analysis, and WGCNA to Identify Septic Shock‐Related Genes

In order to systematically identify core genes associated with septic shock, the present study integrated multiple batch‐derived transcriptomic datasets for a combined analysis. Principal component analysis (PCA) confirmed that, prior to correction for batch effects, samples clustered distinctly according to dataset origin (Figure 1A). After correction, the overlap of samples from different cohorts in the PCA space significantly increased, laying the foundation for subsequent pooled analysis. Differential expression analysis was performed on the integrated cohort (Figure 1B). Volcano plots revealed a large number of significantly differentially expressed genes between the sepsis group and the control group, reflecting the remodeling of immune‐inflammatory and metabolic transcriptional programs under disease conditions (Figure 1C). Heatmaps of differentially expressed genes clearly distinguished the two groups, demonstrating the potential of these genes for disease classification (Figure 1D). We further employed weighted gene co‐expression network analysis (WGCNA) to identify disease‐associated co‐expression modules (Figure 1E). Module‐trait association analysis revealed that modules such as turquoise, blue, and brown exhibited a high degree of correlation with the sepsis or healthy control phenotypes (Figure 1F). Analyses of module membership and gene significance further confirmed the synergistic association between genes within these modules and the disease phenotypes (Figure 1G, H). Finally, the differentially expressed genes and genes from the WGCNA disease‐associated modules were jointly included in the candidate gene set for subsequent screening.

**Figure 1:**
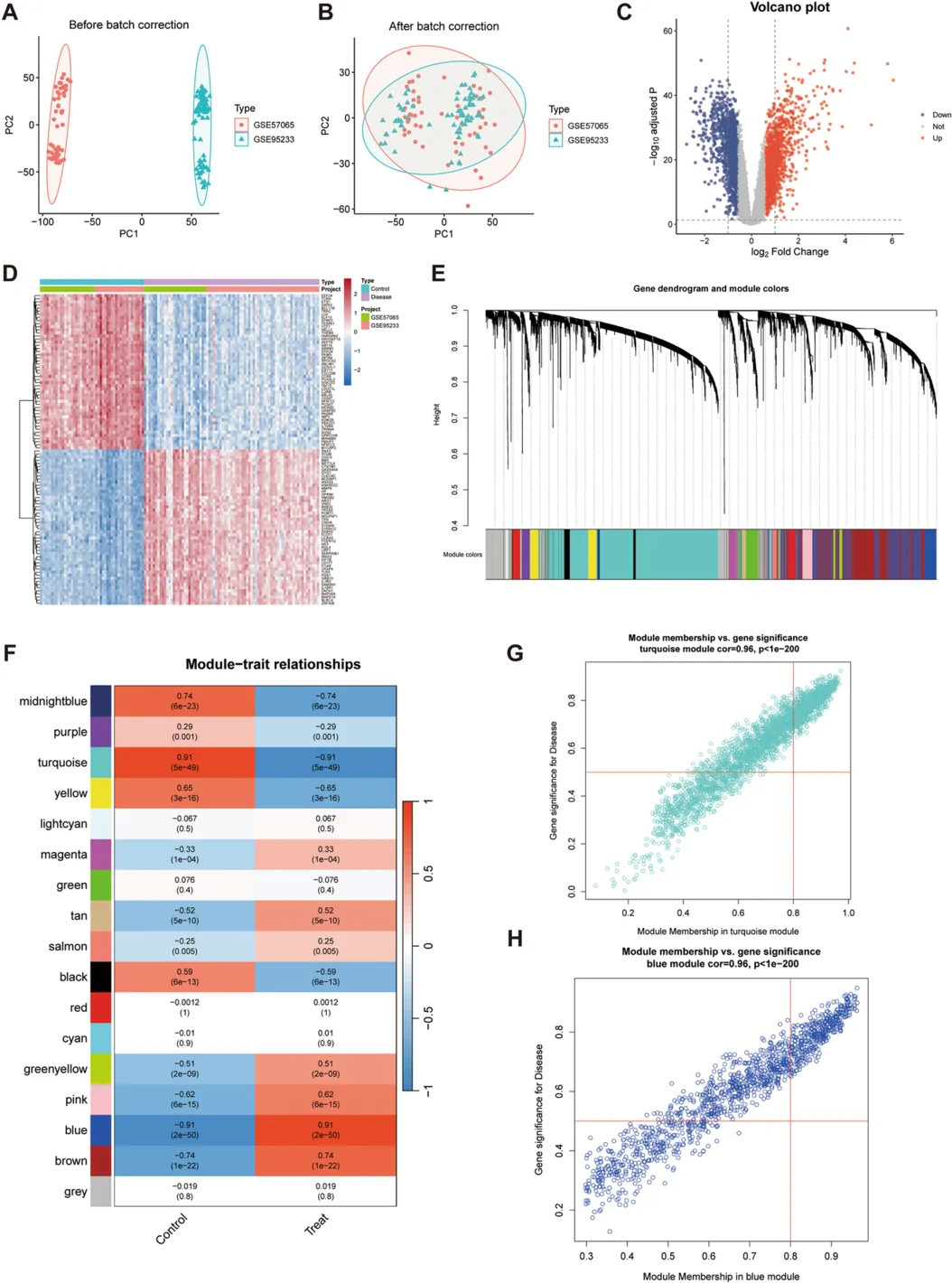
Integrated bulk transcriptome analysis identifies differentially expressed genes and disease‐associated co‐expression modules in septic shock. (A–B) Principal component analysis (PCA) showing sample distribution before and after batch correction of two merged datasets (GSE57065 and GSE95233). Obvious separation between dataset batches was eliminated after batch removal, enabling combined downstream analysis. (C) Volcano plot illustrating global differential gene expression between septic shock patients and healthy controls; red dots denote upregulated genes, blue dots represent downregulated genes, and gray dots indicate non‐significant transcripts. (D) Heatmap of top differentially expressed genes, displaying distinct transcriptional signatures that robustly separate control and septic shock samples. (E) Gene dendrogram and corresponding color‐coded co‐expression modules generated via weighted gene co‐expression network analysis (WGCNA). (F) Heatmap of module‐trait correlation analysis. Each row corresponds to a co‐expression module, while columns represent healthy control and septic shock disease status; correlation coefficients and *p*‐values are annotated within each cell. Turquoise, blue, and brown modules exhibit strong positive correlations with septic shock phenotype. (G–H) Scatter plots of module membership versus gene significance for the turquoise and blue modules, respectively. Significant positive linear correlations confirm that genes with high intramodular connectivity are tightly associated with septic shock.

### 3.2 Candidate Gene Screening and Functional Enrichment Analysis

To further refine the candidate target repertoire, we first integrated target prediction results from three publicly available databases—ChEMBL, PharmMapper, and SwissTargetPrediction—and identified two targets consistently predicted across all three platforms (Figure 2A). Subsequently, intersection analysis was performed across compound‐associated targets, differentially expressed genes (DEGs), and genes from WGCNA‐identified disease‐associated modules, ultimately yielding 46 overlapping candidate genes (Figure 2B).

**Figure 2:**
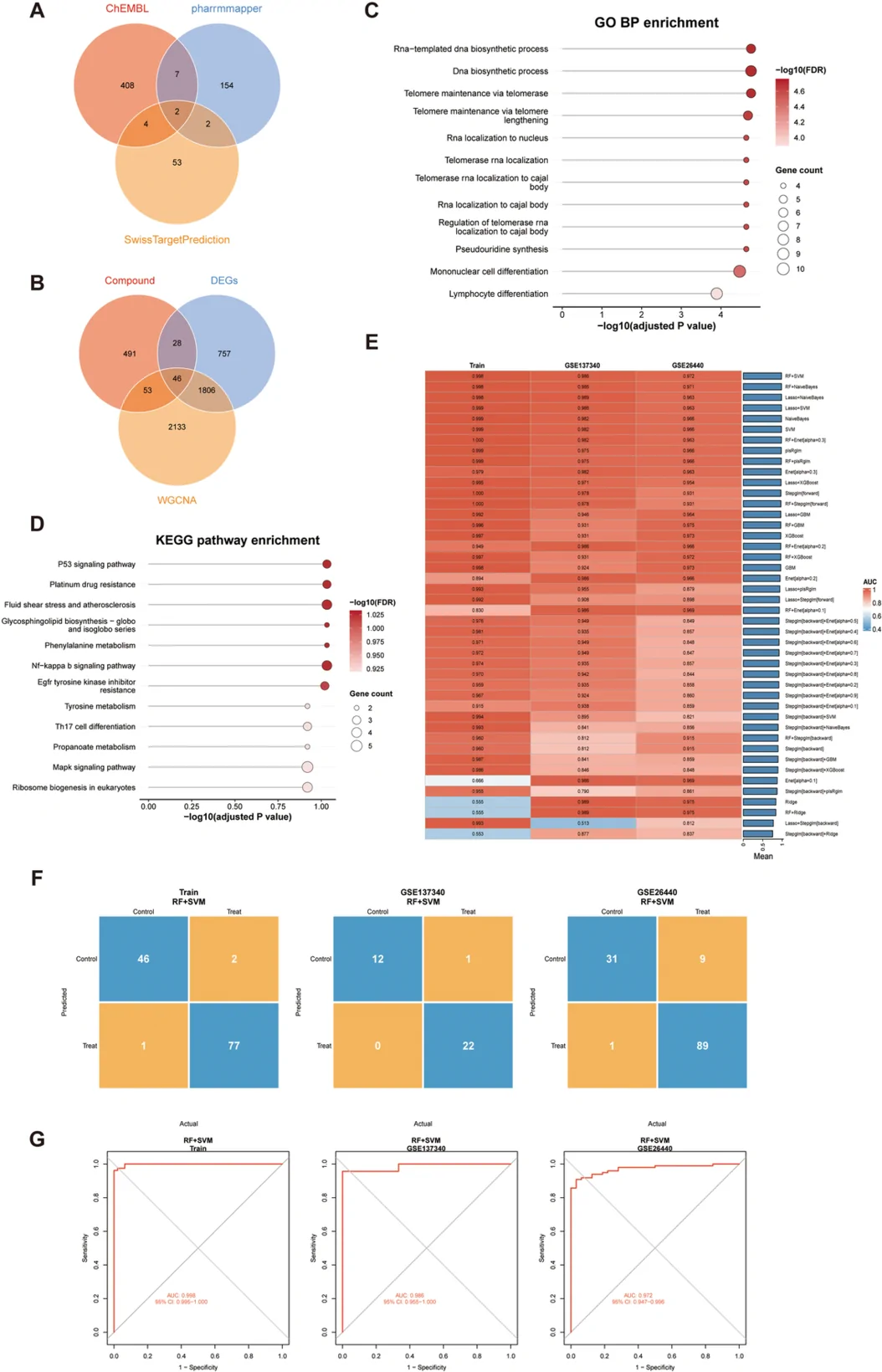
Screening and identification of candidate target genes, functional enrichment analysis, and efficacy verification of machine learning diagnostic models. (A) Venn diagram showing the overlap of putative compound targets predicted by ChEMBL, PharmMapper, and SwissTargetPrediction, with targets shared across all three databases identified. (B) Venn diagram depicting the intersection among compound‐associated targets, differentially expressed genes (DEGs), and weighted gene co‐expression network analysis (WGCNA)‐derived disease module genes, yielding 46 overlapping candidate genes. (C) Bubble plot of GO biological process (BP) enrichment for candidate genes, where bubble size indicates enriched gene count and color gradient denotes adjusted *p*‐value significance. (D) Bubble plot of KEGG pathway enrichment for candidate genes, where bubble size indicates enriched gene count and color gradient denotes false discovery rate (FDR) significance. (E) Heatmaps of feature gene expression profiles across the training set, GSE137340 validation set, and GSE26440 validation set; the adjacent bar plot presents single‐gene diagnostic performance (AUC) for each feature gene. (F) Confusion matrices of the random forest combined with support vector machine (RF + SVM) diagnostic model in the training set, GSE137340, and GSE26440 datasets, demonstrating sample classification accuracy. (G) ROC curves of the RF + SVM model in the training set and two external validation sets, with AUC and 95% confidence intervals annotated, to assess overall diagnostic efficacy and cross‐dataset robustness.

GO Biological Process enrichment analysis revealed that these candidate genes were primarily enriched in processes including RNA‐template‐dependent DNA biosynthesis, DNA biosynthesis, telomerase‐dependent telomere maintenance, RNA nuclear localization, and pseudouridine synthesis, as well as immune‐related biological processes such as monocyte differentiation and lymphocyte differentiation (Figure 2C). Preliminary KEGG pathway analysis indicated that the candidate genes were implicated in the p53 signaling pathway, NF‐κB signaling pathway, Th17 cell differentiation, MAPK signaling pathway, fluid shear stress and atherosclerosis, and a spectrum of metabolism‐related pathways (Figure 2D). Collectively, these findings suggest that the identified candidate genes may coordinately participate in immune cell differentiation, inflammatory signal transduction, cellular stress responses, and metabolic regulation.

### 3.3 Multiple Algorithm‐Based Machine Learning Models Demonstrate Favorable Performance in Identifying Septic Shock

Based on the expression profiles of the identified candidate genes, we systematically evaluated the classification performance of 113 machine learning algorithms and their combinatorial configurations. Model performance was evaluated separately in the training set and two independent external validation sets. The AUC heatmap revealed that multiple models achieved robust classification efficacy across all datasets, among which the RF + SVM combined model exhibited the most stable cross‐dataset performance (Figure 2E).

Confusion matrix analysis showed that the RF + SVM model correctly identified 46 control and 77 septic shock samples in the training set, corresponding to an overall accuracy of 97.6%. In the GSE137340 validation set, 34 of 35 samples were correctly classified, yielding an accuracy of 97.1%. For the GSE26440 validation set, the model correctly classified 120 of 130 samples, with an accuracy of 92.3% (Figure 2F).

Subsequent receiver operating characteristic (ROC) analysis further demonstrated that the RF + SVM model attained an area under the curve (AUC) value of 0.998 in the training set, with values of 0.986 and 0.972 in the GSE137340 and GSE26440 external validation sets, respectively (Figure 2G). Collectively, these results indicate that the established model delivers strong discriminative capacity in the training set and robust generalizability across heterogeneous external datasets.

### 3.4 SHAP Interpretability Analysis Identifies *SORT1* as the Key Feature With the Highest Contribution in the Model

To dissect the contribution of each candidate gene to model prediction performance, we conducted SHAP interpretability analysis on the optimal RF + SVM model. By integrating differential expression profiles and model‐based feature selection, five genes (*SORT1*, *IL18RAP*, *PLEKHA1*, *SLC38A1*, and *LDHA*) were finally identified as core feature genes (Figure 3A).

**Figure 3:**
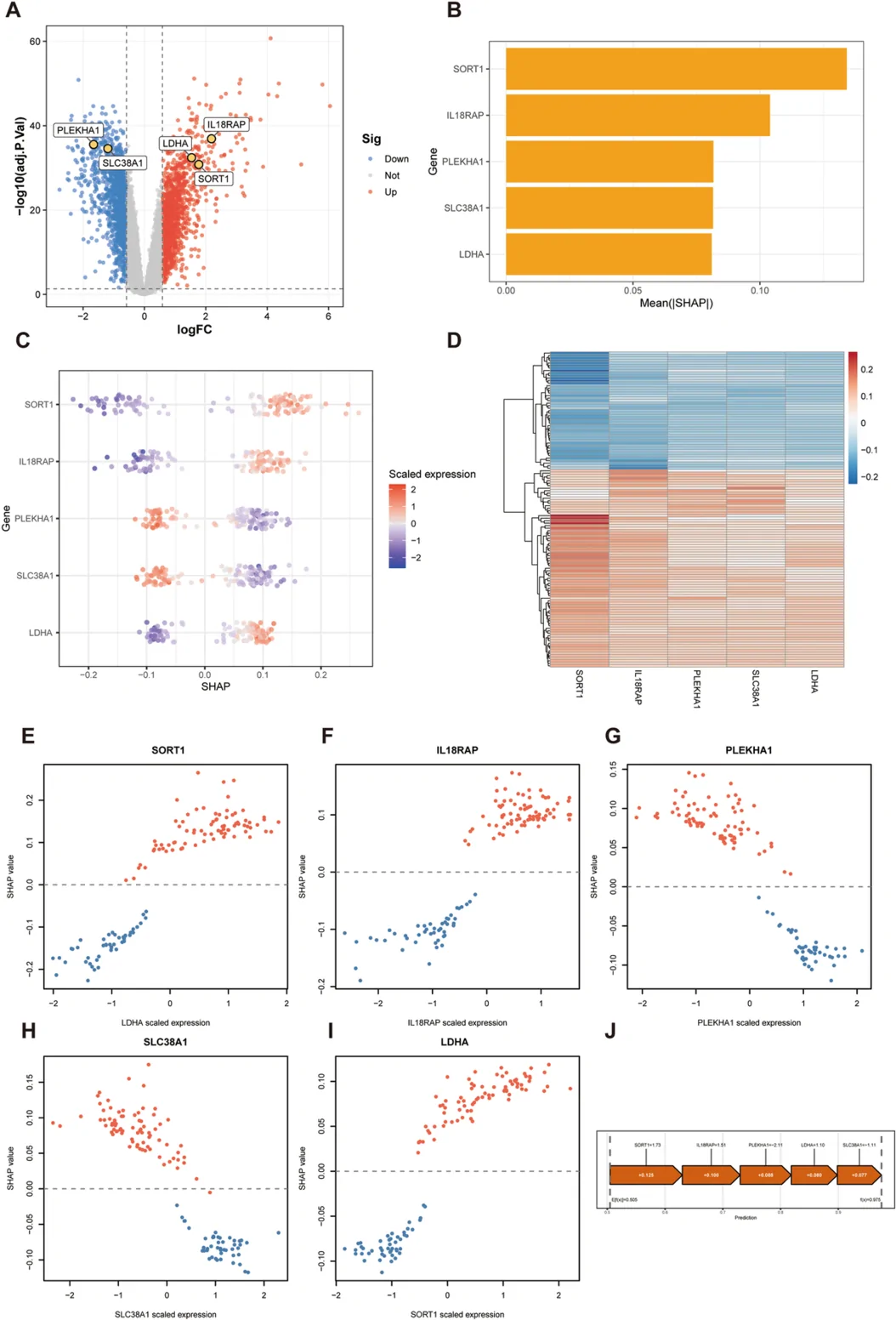
Screening of core diagnostic characteristic genes and interpretability analysis of SHAP model. (A) Volcano plot showing the differential expression of candidate genes. The x‐axis represents log‐transformed fold change (logFC), and the y‐axis represents ‐log10‐adjusted *p* value. Downregulated, non‐significant, and upregulated genes are shown in blue, gray, and red, respectively. The five core feature genes retained in the final model are highlighted. (B) Feature‐importance bar plot based on mean absolute SHAP values, indicating the relative contribution of each core gene to model prediction. (C) SHAP summary beeswarm plot showing the association between standardized gene expression and SHAP values. Dot color represents the standardized expression level, whereas the horizontal distribution reflects the magnitude and direction of each gene's contribution across samples. (D) Sample‐level SHAP heatmap showing the distribution of SHAP values for the five core genes across all samples. Colors indicate the direction and magnitude of gene‐specific contributions to individual predictions. (E‐I) SHAP dependence plots for *SORT1*, *IL18RAP*, *PLEKHA1*, *SLC38A1*, and *LDHA*, respectively, illustrating the relationships between standardized gene expression and corresponding SHAP values. (J) SHAP waterfall plot for a representative sample, showing the positive and negative contributions of individual genes to the final model output.

Feature importance ranking based on mean absolute SHAP values revealed that *SORT1* exerted the strongest contribution to model output, followed by *IL18RAP*, whereas *PLEKHA1*, *SLC38A1*, and *LDHA* also made substantial predictive contributions (Figure 3B). The SHAP summary plot demonstrated that high expression levels of *SORT1*, *IL18RAP*, and *LDHA* were generally associated with positive SHAP values, indicating that upregulation of these genes biased the model toward a septic shock classification. In contrast, high expression of *PLEKHA1* and *SLC38A1* predominantly corresponded to negative SHAP values, whereas their reduced expression promoted classification into the disease group (Figure 3C).

Sample‐level SHAP heatmaps and dependency analyses further corroborated that the predictive contributions of individual genes displayed distinct expression‐dependent patterns and directional effects (Figure 3D‐I). SHAP waterfall plots for representative individual samples consistently indicated that *SORT1* conferred the greatest single‐gene predictive contribution among all core features (Figure 3J). Integrating evidence from differential expression analysis, feature importance ranking, and directional interpretability, *SORT1* was selected as the key candidate gene for subsequent single‐cell spatial localization and mechanistic investigation.

### 3.5 Single‐Cell Transcriptome Analysis Reveals That *SORT1* Is Mainly Localized in CD16+ Monocytes

To delineate the cellular origin of *SORT1* expression across heterogeneous immune cell populations, we further analyzed single‐cell transcriptomic data from the GSE175453 dataset. After stringent quality control, batch effect correction, dimensionality reduction, and unsupervised clustering, a total of 18 cell clusters were identified (Figure 4A). Cell type annotation was performed based on well‐established canonical marker genes, and these clusters were assigned to major immune cell lineages, including CD14+ monocytes, CD16+ monocytes, CD4 + T cells, CD8 + T cells, NK cells, B cells, plasma cells, megakaryocytes, dendritic cells, and hematopoietic stem/progenitor cells (Figure 4B).

**Figure 4:**
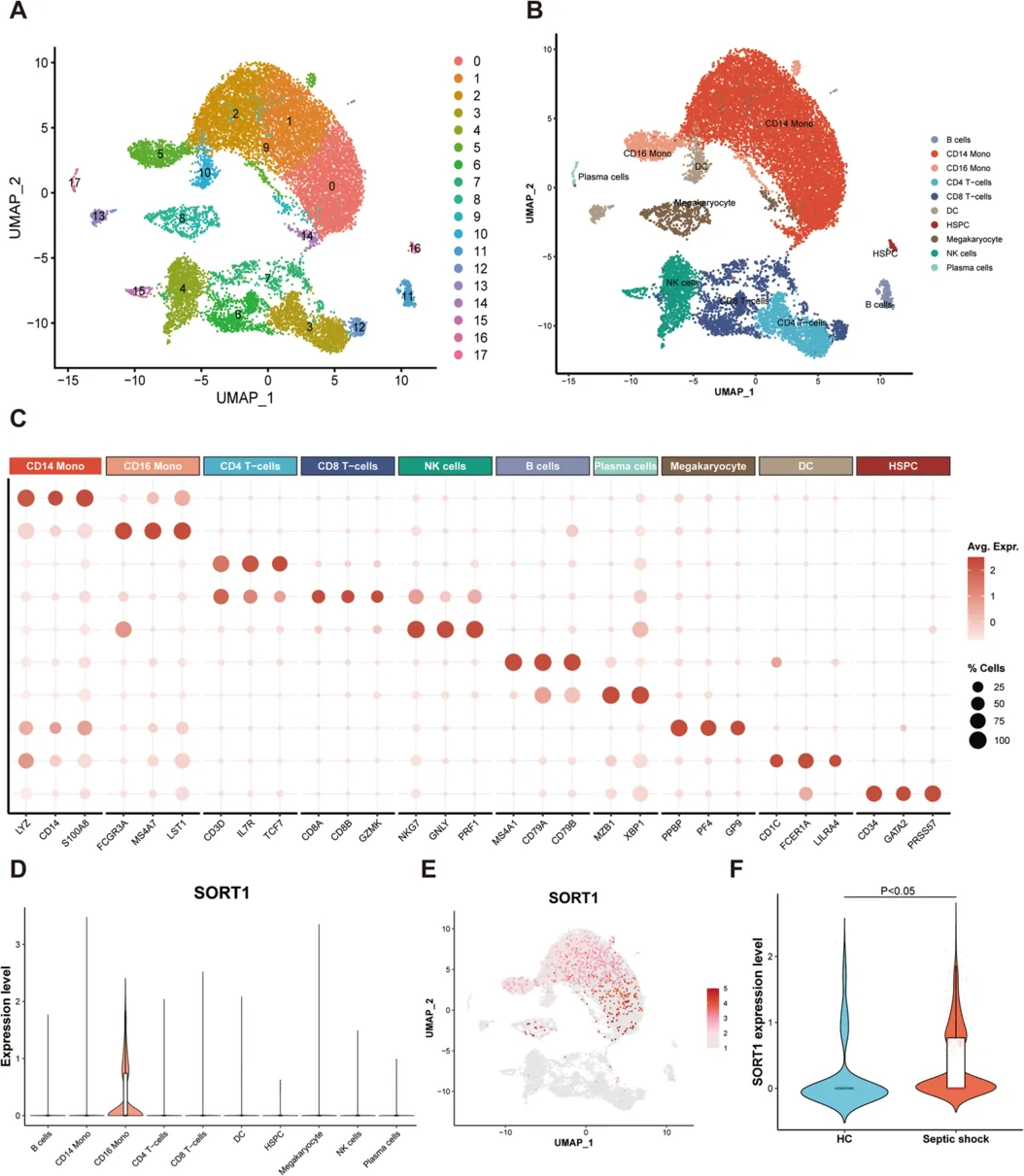
Single‐cell transcriptomic profiling identifies CD16+ monocytes as the predominant cellular source of *SORT1* in septic shock. (A) UMAP visualization of 18 transcriptionally distinct cell clusters. (B) UMAP plot showing the annotation of major immune cell populations. (C) Dot plot of canonical marker genes used for cell‐type annotation. Dot size represents the percentage of marker‐positive cells, and color intensity indicates average expression. (D) Violin plot showing the distribution of *SORT1* expression across the annotated cell populations. (E) UMAP feature plot illustrating the cellular distribution of *SORT1* expression. (F) Comparison of *SORT1* expression in CD16+ monocytes between healthy controls (HC) and patients with septic shock. Statistical significance was assessed using the Wilcoxon rank‐sum test, *p* < 0.05.

Heatmap visualization of canonical marker genes verified that each cell population exhibited gene expression patterns consistent with its known biological properties. For instance, CD14+ monocytes showed high expression of *LYZ*, *CD14*, and *S100A8*; CD16+ monocytes abundantly expressed *FCGR3A*, *MS4A7*, and *LST1*; T cells were characterized by elevated expression of *CD3D*, *IL7R*, or *CD8A/CD8B*; and NK cells highly expressed *NKG7*, *GNLY*, and *PRF1*. These marker expression patterns collectively validated the reliability of the cell type annotation (Figure 4C).

*SORT1* expression displayed prominent cell‐type specificity across distinct immune cell subsets. Violin plots and Uniform Manifold Approximation and Projection (UMAP) feature plots demonstrated that *SORT1* was predominantly expressed in monocyte populations, with particularly marked enrichment in CD16+ monocytes, whereas its expression was relatively low in lymphocytes and other immune cell types (Figure 4D, E). Further differential expression analysis restricted to the CD16+ monocyte subset revealed that *SORT1* expression was significantly higher in the septic shock group than in the healthy control group (*p *< 0.05, Figure 4F). Collectively, these findings suggest that *SORT1* may exert a pivotal function in the functional reprogramming of CD16+ monocytes during septic shock progression.

### 3.6 In Silico Knockout of *SORT1* Significantly Perturbs the Antigen Presentation and Interferon Response Networks in CD16+ Monocytes

To explore the regulatory role of *SORT1* in CD16+ monocytes, we performed *in silico SORT1* knockout in septic shock‐derived CD16+ monocytes using scTenifoldKnk. Ranked by perturbation magnitude, *B2M* and *FTL* were the most significantly disrupted genes, followed by *RPS4Y1*, *CD74*, and multiple HLA family members (Figure 5A, B).

**Figure 5:**
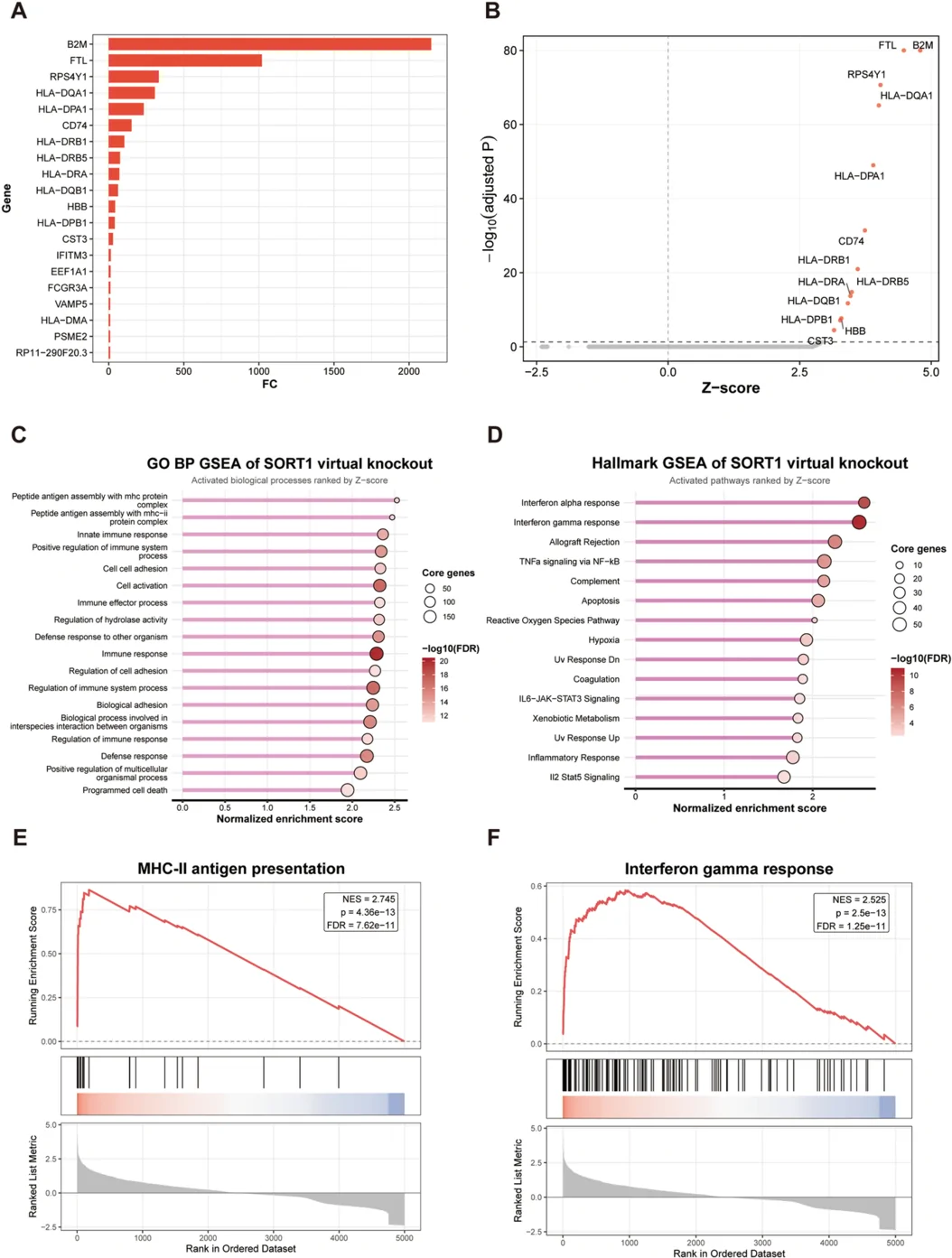
Analysis of Gene Network Perturbations and Functional Enrichment in CD16+ Monocytes Upon In Silico *SORT1* Knockout. (A) Bar plot of the top genes ranked by the magnitude of network perturbation following in silico *SORT1* knockout. (B) Volcano plot showing gene‐level perturbation Z scores and adjusted P values. The most significantly perturbed genes are labeled. (C) GO biological process GSEA of genes ranked by perturbation Z score. Bubble size represents the number of leading‐edge genes, and color intensity indicates the FDR significance. (D) Hallmark GSEA showing the principal signaling pathways enriched after in silico *SORT1* knockout. Plot annotations are as described in panel C. (E) GSEA plot for the MHC class II antigen‐presentation pathway, with normalized enrichment score (NES), P value, and FDR indicated. (F) GSEA plot for the interferon‐γ response pathway, displayed as in panel E.

Most perturbed genes encode major histocompatibility complex (MHC) molecules and core antigen presentation components. CD74 mediates MHC‐II assembly and intracellular trafficking, HLA molecules constitute the MHC‐II presentation system, and B2M supports MHC‐I‐associated antigen presentation. Widespread disruption of these genes indicates that SORT1 is closely involved in antigen processing and presentation in CD16+ monocytes.

GSEA based on Z‐score‐ranked genes showed that perturbed genes were enriched in Gene Ontology biological processes including MHC‐associated antigen peptide assembly, innate immune response, and cell activation (Figure 5C). Hallmark analysis revealed enrichment of interferon responses, TNF‐α/NF‐κB signaling, complement, apoptosis, reactive oxygen species, hypoxia, coagulation, IL‐6/JAK/STAT3 signaling, and inflammatory pathways among upregulated genes after *SORT1* knockout (Figure 5D).

Representative GSEA plots yielded normalized enrichment scores of 2.745 for the MHC‐II antigen presentation pathway (*P* = 4.36 × 10^−^^13^, FDR = 7.62 × 10^−^^11^) and 2.525 for the interferon‐γ response pathway (*P* = 2.50 × 10^−^^13^, FDR = 1.25 × 10^−^^11^) (Figure 5E, F). Collectively, network perturbations induced by *SORT1* knockout concentrate on antigen presentation and interferon‐driven inflammation, suggesting *SORT1* participates in maintaining the immunoregulatory phenotype of CD16+ monocytes in septic shock.

(E) GSEA plot for the MHC class II antigen‐presentation pathway, with normalized enrichment score (NES), *p* value, and FDR indicated. (F) GSEA plot for the interferon‐γ response pathway, displayed as in panel E.

## 4 Discussion

Through integrative analyses of bulk transcriptomics, machine learning, single‐cell transcriptomics, and in silico gene knockout, the present study identified *SORT1* as a potential key regulatory gene associated with septic shock, and further uncovered its putative role in modulating the antigen presentation function of CD16+ monocytes.

Septic shock is a critical syndrome triggered by infection, characterized by uncontrolled systemic inflammation and disrupted immune homeostasis. Its pathogenesis involves complex multi‐dimensional processes, including innate immune activation, adaptive immune suppression, metabolic reprogramming, and endothelial injury [1, 28, 29, 30, 31]. Based on bulk transcriptomic data, our study identified significant transcriptomic differences between septic shock samples and healthy controls. Furthermore, multiple co‐expression modules highly correlated with disease phenotypes were identified via Weighted Gene Co‐expression Network Analysis (WGCNA). By integrating differentially expressed genes, core genes from WGCNA modules, and candidate targets, we screened a panel of candidate genes with both potential diagnostic value and mechanistic significance.

In the process of machine learning‐based feature selection and diagnostic model construction, the gene panel consisting of *SORT1*, *IL18RAP*, *PLEKHA1*, *SLC38A1*, and *LDHA* exhibited excellent diagnostic performance for septic shock and could serve as a core diagnostic signature. Specifically, SHAP interpretability analysis revealed that *SORT1* ranked top in feature contribution, acting as the key predictive feature underpinning the model's accurate discrimination of septic shock status. Distinct from conventional differential expression analysis which only relies on differences in expression abundance, SHAP analysis quantifies and explains the importance of key genes in terms of their predictive weight in the model, providing a more reliable screening basis for subsequent mechanistic investigations centered on core molecules.

Cell clustering analysis of scRNA‐seq further validated the cell‐type‐specific expression pattern of *SORT1*: this gene was predominantly enriched in the CD16+ monocyte subset, instead of being widely expressed across immune cells of all lineages. Accumulating evidence indicates that CD16+ monocytes play pivotal roles in inflammatory response regulation, antigen presentation, directed tissue migration, and immune homeostasis remodeling. In this study, we found that the transcriptional level of *SORT1* in CD16+ monocytes was significantly upregulated in septic shock patients compared with healthy controls, suggesting that *SORT1* may act as a critical regulator involved in monocyte functional reprogramming during the pathological progression of septic shock.

To delineate the potential regulatory function of *SORT1* in CD16+ monocytes, we conducted in silico gene knockout analysis at the single‐cell level via the scTenifoldKnk algorithm based on scRNA‐seq data, to predict the transcriptomic effects of SORT1 knockout. The results demonstrated that the overall number of genes with significantly perturbed transcription levels after *SORT1* knockout was relatively small, yet their functions were highly concentrated, with core perturbed molecules including *B2M*, *CD74*, and multiple HLA class II family members. This expression pattern indicates that the regulatory effect of *SORT1* is not pervasive across the whole transcriptome, but selectively modulates the immune functional module associated with antigen presentation. Consistently, subsequent GSEA further corroborated this finding: pathways related to antigen processing and presentation, MHC class II‐dependent antigen presentation, and interferon response were all significantly enriched, providing pathway‐level evidence supporting the hypothesis that *SORT1* regulates the antigen presentation function of monocytes.

It should be noted that the differentially perturbed genes including *RPS4Y1* and *HBB* identified in this in silico gene knockout analysis are prone to interference from confounding factors such as sample sex differences and hemoglobin‐related background signals. Accordingly, these findings should be interpreted with caution and should not be regarded as core mechanistic targets of *SORT1* regulatory function. In comparison, the antigen presentation functional module consisting of *CD74*, *HLA‐DRA*, *HLA‐DRB1*, *HLA‐DQA1*, *HLA‐DPA1*, *HLA‐DPB1*, and *B2M* demonstrates high biological consistency and functional coherence at the pathway level, which better reflects the authentic regulatory effect of *SORT1* and serves as the core functional axis mediating functional state remodeling of CD16+ monocytes.

Several limitations of the present study should be noted and warrant further investigation in future work. First, both the machine learning‐based screening and single‐cell transcriptomic profiling were performed using retrospective datasets from publicly available databases. Given inherent constraints in sample source and clinical heterogeneity, our findings need to be further validated in large‐scale, multicenter prospective clinical cohorts. Second, scTenifoldKnk represents an in silico gene knockout strategy at the computational simulation level; its results only enable inference of potential transcriptional regulatory relationships and cannot fully substitute the validation power of loss‐of‐function experiments. For future work, the regulatory role of *SORT1* in the *B2M*/*CD74*/HLA‐II antigen presentation module can be systematically verified through siRNA‐mediated transient knockdown, CRISPR‐Cas9‐mediated stable knockout, and in vitro inflammatory stimulation models of monocytes. Furthermore, the specific molecular pathway underlying *SORT1*‐mediated regulation of antigen presentation remains undefined to date. Whether direct protein‐protein interactions exist between *SORT1* and the antigen presentation machinery still requires in‐depth exploration via molecular biological assays. No additional experimental validation was performed in the present revision because our group currently lacks the necessary experimental facilities and funding. Further experimental work is planned when the required resources become available.

Taken together, based on integrated multi‐omics analyses, our study puts forward a putative mechanistic model of monocyte immune remodeling in septic shock. During the pathological progression of septic shock, *SORT1* expression is markedly elevated in the CD16+ monocyte subset, which may mediate monocyte immune functional reprogramming by targeting the *B2M*/*CD74*/HLA‐mediated antigen presentation functional module and interfering with interferon‐related inflammatory response pathways. The *SORT1* identified herein not only serves as a novel candidate molecular biomarker for the early diagnosis of septic shock, but also provides a brand‐new research target and entry point for deciphering the regulatory mechanism underlying sepsis‐associated immune dysfunction.

## 5 Conclusion

By integrating multi‐omics analysis with machine learning algorithms, this study identified *SORT1* as a pivotal gene associated with septic shock. Single‐cell transcriptomic profiling confirmed that *SORT1* is predominantly enriched in the CD16+ monocyte subset, with its expression significantly upregulated under septic shock conditions. In silico gene knockout and GSEA suggested that *SORT1* may participate in the immune regulation of CD16+ monocytes by modulating the *B2M/CD74/HLA*‐mediated antigen presentation module and interferon response pathways. Collectively, our findings provide a novel candidate target and research direction for uncovering the molecular mechanism underlying monocyte immune remodeling in septic shock.

## Declaration of Generative AI and AI‐Assisted Technologies in the Writing Process

The authors declare that no generative AI or AI‐assisted software was employed to conduct data analysis, interpret clinical findings, or draft the core medical hypotheses and concepts presented in this manuscript. AI‐assisted tools were used during revision for language editing and formatting.

## Author Contributions

**Shulu Wu:** conceptualization, writing – original draft, methodology, writing – review and editing, formal analysis, software, data curation, resources. **Jiaying Zhang:** methodology, supervision, data curation. **Shiqi Guo:** methodology. **Yuting Hu:** methodology. **Hui Shen:** methodology. **Yingjie Xu:** methodology. **Bining Yang:** conceptualization, supervision, writing – review and editing, methodology.

## Funding

The authors have nothing to report.

## Ethics Statement

The authors have nothing to report.

## Conflicts of Interest

The authors declare no conflicts of interest.

## Supporting information


Supporting File

## Data Availability

The raw bulk and single‐cell RNA‐seq data supporting the findings of this study are accessible from the NCBI GEO database (accessions: GSE57065, GSE95233, GSE26440, GSE137340, GSE175453). Further analysis scripts are available from the corresponding author.
